# Composition and Diversity of Soil Microbial Community Associated With Land Use Types in the Agro–Pastoral Area in the Upper Yellow River Basin

**DOI:** 10.3389/fpls.2022.819661

**Published:** 2022-04-25

**Authors:** Shiliang Liu, Yongxiu Sun, Fangning Shi, Yixuan Liu, Fangfang Wang, Shikui Dong, Mingqi Li

**Affiliations:** ^1^State Key Laboratory of Water Environment Simulation, School of Environment, Beijing Normal University, Beijing, China; ^2^School of Grassland Science, Beijing Forestry University, Beijing, China

**Keywords:** microbial community composition, soil microbial diversity, agro–pastoral area, soil property, spatial distribution

## Abstract

The microorganisms of soil are sensitive to their living microenvironment, and their community structure and function will change with the environmental conditions. In the agro–pastoral area on the Qinghai–Tibet Plateau, revealing the diversity of the soil microbial communities and its response to different soil physicochemical properties and environmental factors are important for ecosystem management. The microbial (bacteria and archaea) community composition and diversity under different land use types (cultivated land, grazing grassland and planted forest) were analyzed by 16S rRNA (V4 region) method in a typical agro–pastoral region in the upper Yellow River basin. Also, the soil nutrients were studied and correlated with the microbial community. The results showed that the soil nutrient contents in grassland were low, but the available nutrients were relatively high. There was a great spatial variability under different distances to the river. The microbial community diversity was lower in the grassland than the cultivated land and forest land closer to the river. For all land uses, the dominant phyla of soil microorganisms included Proteobacteria, Actinobacteria, and Bacteroidetes, while the abundance of Clostridia was significantly higher than that of the other groups, indicating that Clostridia dominated the Firmicutes and affected soil microbial community composition. The linear discriminant analysis (LDA) effect size (LefSe) analysis showed different biomarkers were more abundant in grassland than other land use types, suggesting that the structure and diversity of soil microorganisms in grassland were significantly different compared with cultivated land and forest land. The distance-based redundancy analysis (db-RDA) results showed that the total phosphorus (TP) and calcium (Ca) were the key environmental factors affecting the diversity and abundance of the soil microbial community in cultivated land and forestland, respectively. However, the microbial diversity in grassland was more related to spatial distance of the river. These results provided a theoretical basis for the changes in the composition, structure, and function of soil microbial communities in agro–pastoral areas.

## Introduction

The land use contains a variety of activities that exploit the soil resources to achieve the desired economic or social purposes of humans, which has an important impact on soil ecosystems (Yang et al., 2017). The different land use types are the result of the interaction between human activities and natural factors, which can cause soil-related ecological effects (Ji et al., 2020). The land use changes bring great influence on soil physical and chemical properties, soil biodiversity, and soil ecological functions (Ji et al., 2020; Zhang et al., 2020; Ma et al., 2021). The rational land use management can improve soil quality and enhance the resistance to external disturbance, while unreasonable mode can lead to a sharp decrease in soil biodiversity and degradation of ecological functions, thus affecting the sustainable development of soil resources (Yao et al., 2000; Lal, 2004). Therefore, the research on the effects of the different land use patterns on soil biological communities is a hotspot in the current climate and the environmental change research (Yang et al., 2017; Li, 2021; Ma et al., 2021).

The soil microorganism plays an important role in soil biochemistry, which is involved in the decomposition of soil organic matter, humus formation, and nutrient cycling. At the same time, soil microorganism is critical to maintaining ecosystem function as the connection between soil and plants (Chen et al., 2019). Therefore, the microbial diversity is widely used to evaluate soil fertility and land quality (Lamb et al., 2011; Barberan et al., 2012; Fierer et al., 2012; Berthrong et al., 2013; Lauber et al., 2013). The studies have shown that soil microorganisms are more capable of sensitive changes in the environment than plants and animals (Willig et al., 2003; Qian and Ricklefs, 2004; Zhou et al., 2008; Zhang et al., 2013). As one of the important groups of the soil microorganisms, the soil bacteria can adapt to the land use changes by adjusting the community structure and diversity level, which may play a key role in the ecosystem’s function (Yang et al., 2017; Li, 2021). Land use changes may alter soil nutrient cycling, thereby affecting soil biome diversity (Wagg et al., 2019). Understanding the information of microbial structure and functional diversity is of great significance to clarify the role of microbial communities in different land use types, thus can provide theoretical guidance for evaluating and optimizing soil quality and land use management. The traditional methods of bacterial culture and identification are complex and most microorganisms cannot be cultured in the laboratory, which cannot satisfy the macroscopic view of exploring the diversity of bacterial communities in the whole microecosystem (Lou et al., 2014). With the development of next-generation sequencing and the development of molecular biology comprehensive analysis, the method of sequencing analysis using conserved section specific amplification V3–V4 16S rRNA has been widely used in the recent years (Liu et al., 2020).

Qinghai Province is located in the inland of Northwest China, with abundant land resources and water resources. The upper reaches of the Yellow River in Hainan Tibetan Autonomous Prefecture have typical geographical conditions and sufficient water sources, and the main vegetation types include steppe, forest, shrub, alpine shrub, and alpine meadow. Grassland, cultivated land, and forest land are the main land use types. Therefore, this agro–pastoral area forms a production pattern relying on agriculture and supplemented by animal husbandry, which is vital to the local socio–economic development. However, the influence of natural factors and human activities has caused great changes in landscape pattern. The transformation of natural vegetation, especially grassland to farmland, has not only caused the change of landscape pattern but also brought great pressure to the ecosystem (Lv et al., 2005). These land use changes alter the physicochemical properties, biological properties, and environmental conditions of soil; thus, significantly affecting the diversity and composition of soil microbial communities (Brockett et al., 2012; Kim et al., 2014). The change of soil microbial community structure can be used as a measure of soil quality, and it can also indicate the change of ecosystem structure and function (Avidano et al., 2005; Rousk et al., 2010; Huang et al., 2014). The agro–pastoral areas are closely related to the economic development and ecological protection, and have high research value. However, most of the studies on the Qinghai–Tibet Plateau focus on grassland and other natural ecosystems, and there are few studies on the compound ecosystem of agricultural and pastoral areas.

At present, many studies have elucidated the diversity of soil microbial community and its relationship with soil physicochemical properties (van Meeteren et al., 2008; Zhang et al., 2013). However, because of the spatial variability of soil microbial community, it is very challenging to study soil microbial community structure at different spatial scales (Amann et al., 1995; Ettema and Wardle, 2002; Ellis et al., 2003; O’Brien et al., 2016). It is well known that the composition of microbial community is affected by light, precipitation, temperature, soil nutrients, and other factors, and these environmental factors are closely related to the geospatial pattern (Romaniuk et al., 2011; Karimi et al., 2017; Seena et al., 2019). Many studies have confirmed that the community composition and diversity of microorganisms are distributed vary with certain variables at spatial scale, such as depth and latitude (Li et al., 2017). To explore the spatial distribution pattern of microorganisms at regional scale, some studies evaluated the diversity and abundance of operational taxonomic units (OTUs) in different water bodies (Djurhuus et al., 2017), altitudes (Zhang et al., 2020), tillage measurements (Wagg et al., 2019), and other environmental conditions. However, few studies analyze soil microbial composition and structure by combining different environmental conditions and different regional scales. Exploring the key driving factors of soil microbial diversity and distribution on the Qinghai–Tibet Plateau will help to deepen the understanding of soil microbial and ecosystem functions.

Accordingly, based on 16S rRNA gene sequencing analysis, this study compared the spatial distribution of microorganisms and their related environmental factors under different land uses in the upper Yellow River basin. The objectives of the present study were as follows: (1) To investigate the response of soil microbial diversity to different land use and different distance gradients to the river; (2) to reveal the spatial distribution of soil microbial community composition; and (3) to assess the effects of soil nutrients on microbial communities. This study will provide a theoretical basis for the change and spatial distribution of soil microbial community diversity in agro–pastoral areas, and has important practical significance for the comprehensive analysis of soil microbial diversity and the maintenance of sustainable land use management.

## Materials and Methods

### Study Sites

**FIGURE 1:**
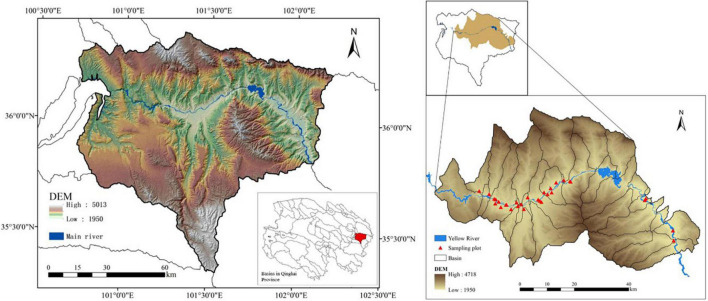
Study area and sampling site.

This study was conducted in the southeast of Hainan Tibetan Autonomous Prefecture in Qinghai Province, including Guide County, Jianzha County, and parts of Guinan County, China. All the sampling sites were located in the gorge section of the Yellow River, an agro–pastoral area, according to the division of the river basin (100°43′ E–102°12′ E; 35°19′ N–36°24′ N) (Figure 1). The area has a typical plateau continental climate, with long sunshine duration time and strong solar radiation. The annual average temperature is 7.2°C and the average annual precipitation is 250–400 mm. The region has multi-level river terraces and hilly landforms in the basin, and contains abundant water resources. The altitude ranges from 1950 to 4718, with high in the north and south, and low in the middle. The land use types include grassland, cultivated land, forestland, bare land, water body, and construction land. Grassland resources are very abundant, and grazing pastures are large in area and widely distributed. The forestland is mainly planted forest. Due to the surrounding mountains along the river, the study area has a warm climate and fertile land. Therefore, most areas have become agricultural production bases, and the main crops are wheat (*Triticum aestivum* L.), highland barley (*Hordeum vulgare* L. var. nudum Hook.f.), and rape plants (*Brassica napus* L.). The cultivated land management is mainly based on crop rotation and intercropping.

### Soil Sample Collection and Experimental Design

The sampling time began in July 2019, and 30 sites were set up along the Yellow River in the study area, with different distance gradients from 100 to 200 m (C1/G1/T1) and 300 to 500 m (C2/G2/T2) to the river. A total of 101 plots were taken from the cultivated land (C), grazing grassland (G), and plantation forest (T). In each plot, three quadrats (5 m × 5 m) were randomly selected in each land use type, and three topsoil (0∼10 cm) samples were randomly collected from each quadrat and mixed into one sample replicate. A total of 303 soil samples were collected. The main crop of sampling sites in the cultivated land is wheat and the main species in grazing grassland include *Polygonum, Aster*, *Carex*, *Carum carvi* L., *Geranium*, *Poaceae*, etc. Forests are mainly cold and warm coniferous forests, and the dominant tree species are *Sabina przewalskii*, *Picea crassifolia*, *Populus cathayana*, *Betula platyphylla*, *Hippophae rhamnoides*, etc. For each sample, one part was naturally air-dried at room temperature for 15 days and sieved (0.2 mm) to remove impurities such as stones and plant roots for soil physicochemical properties analysis, the other part was placed in a self-sealed sterile bag and frozen stored in −4°C for microbial diversity determination. The sample layout is shown in Figure 1.

### Analysis of Soil Properties

The measured soil parameters mainly included the total contents of nitrogen (TN) and carbon (TC), as well as the total phosphorus (TP), total potassium (TK), available phosphorus (AP), available potassium (AK), and calcium (Ca). We weighed 30-mg grinded and screened soil samples, and wrapped them in tin capsules. The TC and TN were determined by Euro EA3000 elemental analyzer. A 50-ml 0.01 M CaCl_2_ was used to extract 5 g of soil samples (Guo and Zhou, 2021). The solution was shaken by an oscillator for 1 h, and precipitated for 0.5 h after shaking (Liu et al., 2021). The soil suspension was filtered by filter paper, and TP, TK, AP, AK, and Ca were determined by ICP (SPECTRO ARCOS, Germany) (Guo and Zhou, 2021; Liu et al., 2021).

### Microbial Diversity Analysis

The total community DNA (TC-DNA) of the samples was extracted by CTAB/SDS method, and the purity and concentration of DNA were detected by agarose gel electrophoresis. The sample DNA was diluted to 1 ng/μl with sterile water. The diluted TC-DNA was used as a template for polymerase chain reaction (PCR), and the V4 region of 16S rRNA was amplified with the specific barcoded primers 515F/806R (515F: 5′-GTGCCAGCMGCCGCGG-3′; 806R: 5′-GGACTACHVGGGTWTCTAAT-3′) (Jing et al., 2015; Chu et al., 2016). Phusion^®^ High-Fidelity PCR Master Mix with GC Buffer (New England Biolabs, Ipswich, MA, United States) and high-fidelity enzyme were used for PCR reaction to ensure efficiency and accuracy of amplification. The PCR products were detected and purified by electrophoresis with 2% agarose gel. The GeneJET purification kit (Thermo Scientific, Waltham, MA, United States) was used for the purification of the product. The library was constructed using Ion Plus Fragment Library Kit 48 rxns. After the constructed library was qualified by Qubit quantification and library test, Ion S5TMXL (Thermo Fisher Scientific, United States) was used for gene sequencing in the IonS5TMXL sequencing platform.

The Cutadapt^1^ (Version 1.9.1; Asshauer et al., 2015) was used to cut the low-quality parts of the reads, and the sample data were separated from the obtained reads according to the Barcode. Afterward, primer sequences were removed and the raw reads were obtained through preliminary quality control. The chimeric sequences were removed by the ultra-fast sequence analysis (Usearch) software to obtain the final effective clean reads (Rognes et al., 2016). The Uparse software^2^ (Version 7.0.1001; Haas et al., 2011) was used to cluster all clean reads sequences into OTUs with 97% identity, and the sequence with the highest occurrence frequency was selected as the representative sequence of OTUs according to the algorithm principle. The species annotation analysis of OTUs sequences was carried out by Mothur method and the SSUrRNA database (Wang et al., 2007) of SILVA132^3^ (set thresholds of 0.8 ∼ 1; Edgar, 2013), then the taxonomic information was obtained and the community composition of each sample was counted at each classification level as follows: kingdom, phylum, class, order, family, genus, and species. The MUSCLE software^4^ (Version 3.8.31; Quast et al., 2013) was used to carry out fast multi-sequence alignment to obtain the phylogeny of all OTUs sequences. Finally, the subsequent α-diversity analysis and β-diversity analysis were all based on the homogenized data.

### Statistical Analyses

The variance analysis was performed using IBM SPSS Statistics (version 22.0.0.0). Alpha-diversity (α-diversity) indices, such as Observed OTUs, Chao1, Shannon, Simpson, ACE, and coverage index, were calculated with Qiime software (Version 1.9.1). For β-diversity (β-diversity) analyses, the weighted unifrac distance was calculated by Qiime software (Version 1.9.1) using evolutionary information between microbial sequences in each sample (Lozupone and Knight, 2005; Lozupone et al., 2011). The WGCNA, stats, and ggplot2 software packages of R software were used for the principal coordinate analysis (PCoA) with weighted unifrac distance. The abundance information of OTUs was used to further construct weighted unifrac distance (Lozupone et al., 2007), which was used to draw the heatmap about β-diversity. The Sklearn package in R software was used for the linear discriminant analysis (LDA). Meanwhile, the analysis of molecular variance (AMOVA) was used to analyze the differences between groups by Mothur software (Roewer et al., 1996). The vegan package in R software was used for the distance-based redundancy analysis (db-RDA) based on the Bray–Curtis distance matrix.

One-way analysis of variance (ANOVA) was used to compare the differences in soil nutrients, soil microbial α-diversity, β-diversity, and relative abundances of bacteria and archaea under the three land use types. Duncan’s multiple range test was used to test the significance of differences between treatments. The 10 phyla and class most abundant were selected to generate a cumulative histogram and the 35 genera most abundant were selected to generate a heatmap. To study the species with significant differences between groups, we used the LDA for effect size (LefSe) to find the biomarkers between groups, and set the filter value of LDA Score to 4.0 (Segata et al., 2011). The results of the LefSe include three parts as follows: LDA score histogram, cladogram, and biomarker abundance comparison histogram in different groups. The method was used to identify biomarkers that were statistically different between groups (Segata et al., 2011; Wang et al., 2020). The AMOVA analysis test the significance of differences between different land use types by the sum of squared deviations (SSD), *F*-test value, and *p-*value. The RDA analysis reflects the Euclidean distance between sample squares on the graph, while db-RDA is a distance-based redundant analysis, which is applicable to any distance matrix. The db-RDA analysis was mainly used to reflect the relationship between microbial communities and environmental factors (Clarke and Ainsworth, 1993). Therefore, db-RDA analysis was used to explore the relationship between microbial community structure and soil nutrients properties.

## Results

### Soil Nutrients and Gene Sequence Characteristics of Soil Samples Under Different Land Use Patterns

The soil nutrients under different land uses and different distances to river showed variations (Table 1). The contents of Ca and TN in cultivated land and forestland were slightly higher than those in grassland. The contents of TC were the highest in forest land, followed by cultivated land, and the contents of TN in grassland were significantly lower than those in the other two land use types (*p* < 0.05), indicating that the soil nutrients of the grassland were relatively low. The contents of TP in cultivated land were significantly higher than those in forestland and grassland (*p* < 0.05), but the content of AP was reverse. Although the difference of AK content between different sample groups was not significant, it was relatively high in grassland, followed by forestland and cultivated land. The difference contents between available nutrients and total nutrients in different land uses indicated that, although the content of TP and TK in cultivated land was higher than grassland and forestland, the available nutrients for plant absorption and utilization were less.

**TABLE 1:** Soil nutrients in each sample group.

	TN [%]	TC [%]	Ca (g/kg)	TK (g/kg)	TP (g/kg)	AK (mg/kg)	AP (mg/kg)
C1	0.089 ± 0.007b	2.070 ± 0.082ab	43.306 ± 1.252b	20.321 ± 0.336b	1.027 ± 0.041b	201.376 ± 37.902a	0.520 ± 0.110a
C2	0.077 ± 0.006b	1.929 ± 0.091ab	41.055 ± 1.456b	19.938 ± 0.313b	0.929 ± 0.044b	177.950 ± 24.158a	0.773 ± 0.306a
G1	0.019 ± 0.006a	1.136 ± 0.119a	35.950 ± 2.530ab	19.088 ± 0.713b	0.556 ± 0.015a	305.770 ± 27.921a	3.275 ± 1.154b
G2	0.030 ± 0.001a	1.160 ± 0.026a	30.828 ± 1.274a	17.039 ± 0.340a	0.507 ± 0.021a	298.546 ± 40.346a	0.565 ± 0.142a
T1	0.113 ± 0.021b	2.751 ± 0.333b	45.963 ± 2.755b	19.880 ± 0.309b	0.654 ± 0.017a	271.065 ± 23.086a	1.305 ± 0.212ab
T2	0.089 ± 0.011b	2.407 ± 0.220b	44.346 ± 2.845b	19.622 ± 0.268b	0.654 ± 0.017a	253.125 ± 25.683a	1.285 ± 0.401ab

*All values are reported as “mean ± standard deviation” based on measurement results for triplicated samples; TN, total nitrogen; TC, total carbon; Ca, calcium; TK, total potassium; TP, total phosphorus; AK, available potassium; AP, available phosphorus; values in the same column without shared lowercases letters mean significant difference at p < 0.05 among the samples. C1: cultivated land with distance to river from 100 to 200 m; C2: cultivated land with distance to river from 300 to 500 m; G1: grassland with distance to river from 100 to 200 m; G2: grassland with distance to river from 300 to 500 m; T1: forestland with distance to river from 100 to 200 m; T2: forestland with distance to river from 300 to 500 m.*

With the increase of distance to the river, among which the difference was significant in the content of TK and AP in grassland (*p* < 0.05). The content of AP in the samples far from the river was 0.565 mg/kg, and increased to 3.275 mg/kg in the samples closer to the river. This indicated that the influence of water on soil nutrients of grassland was higher than that of cultivated land and forest land.

Based on the IonS5TMXL sequencing platform, the single-end sequencing method was used to construct a small fragment library, and the quality control efficiency reached 94.36%. The sequence was clustered into OTUs with 97% identity, and a total of 5985 OTUs were obtained. Supplementary Figure 1 showed the OTUs Venn diagram of different land uses under different distance gradients. According to Supplementary Figure 1, the unique OTUs detected in the three land uses are 747 (C), 845 (T), and 998 (G), respectively. Among them, 3377 common OTUs were found in the six soil samples, accounting for 56.6% of the total OTUs, indicating that the soil microbial composition of cultivated land, forestland, and grassland ecosystems had certain similarity at the OTU level. According to the different distance gradients, the number of unique OUT in cultivated land was 390 (C1) and 357 (C2); the grassland was 824 (G1) and 174 (G2); and the forestland was 394 (T1) and 451 (T2), indicating that the distance from the river affects the species community composition in different ecosystems, among which the grassland ecosystem is obviously affected.

### Diversity Characteristics of Soil Microbial Communities Under Different Land Use Patterns

Alpha diversity can reflect the richness and diversity of the microbial community within the sample (Table 2). The coverage is above 0.98, indicating that the sample library has a high coverage of species, and the sequencing results can represent the real situation of microorganisms in the sample. Along with different distances to the river, the Simpson index of soil components did not change significantly, indicating that the uniformity of soil microbial community did not differ significantly. However, the results showed that the Shannon index and OTUs of G1 group were 8.52 and 2258 (Table 2), respectively, lower than those of other groups, which indicated that the complexity and diversity of the microbial community in the grassland were lower closer to the river. The Chao1 index and ACE index of grassland soil samples were 2571.72 (G1), 2744.9 (G2); and 2642.06 (G1), 2797.38 (G2), respectively, which were significantly lower than those of the other two land uses, indicating that the species richness of soil microorganisms in grassland was less. According to the α-diversity indices, the order of microbial community diversity and richness of the three land use types was as follows: cultivated land > forestland > grassland. For the cultivated land and forest land, the microbial diversity was low in the areas far from the river, while grassland was the opposite. The complexity of microbial community in grassland soil is relatively low, and the closer to the river, there is a tendency to decrease.

**TABLE 2:** Alpha diversity indices of soil microbial communities in different soil samples.

Group	Coverage	Observed OTUs	Shannon	Simpson	Chao1	ACE
C1	0.985	2825	9.52 ± 0.11	0.995 ± 0.001	3139.89 ± 66.09	3253.28 ± 73.35
C2	0.986	2700	9.31 ± 0.17	0.992 ± 0.003	3023.70 ± 101.93	3131.56 ± 110.24
G1	0.987	2258	8.52 ± 0.25	0.987 ± 0.003	2571.72 ± 167.94	2642.06 ± 168.90
G2	0.987	2435	9.16 ± 0.12	0.994 ± 0.001	2744.90 ± 194.26	2797.38 ± 202.87
T1	0.985	2748	9.46 ± 0.09	0.995 ± 0.001	3096.86 ± 63.17	3175.44 ± 64.98
T2	0.985	2673	9.22 ± 0.17	0.993 ± 0.002	3030.65 ± 95.97	3139.05 ± 94.63

*All values are reported as “mean ± standard deviation” based on measurement results for three replicates. C1: cultivated land with distance to river from 100 to 200 m; C2: cultivated land with distance to river from 300 to 500 m; G1: grassland with distance to river from 100 to 200 m; G2: grassland with distance to river from 300 to 500 m; T1: forestland with distance to river from 100 to 200 m; T2: forestland with distance to river from 300 to 500 m.*

**FIGURE 2:**
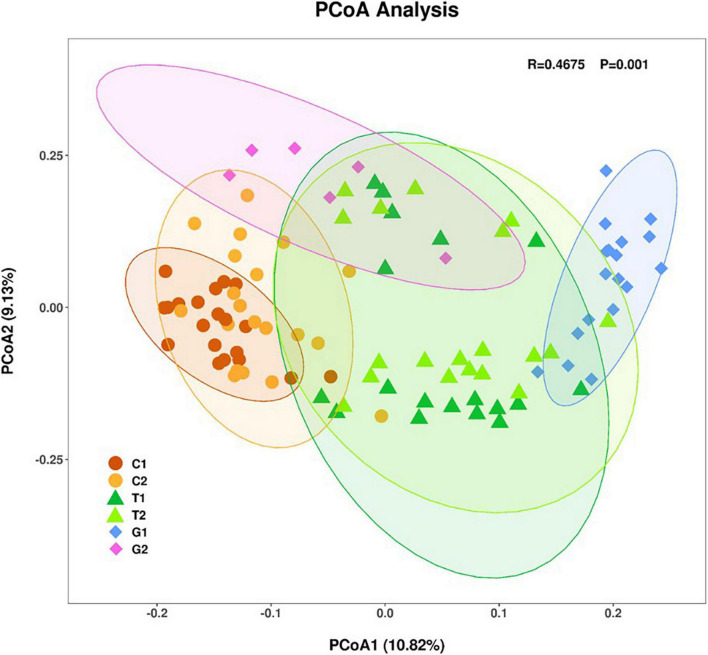
Principal coordinate analysis (PCoA) analysis of sampling points based on unweighted unifrac distances of microbial communities. Each point represented a sample, the same color was the same sampling area.

The β-diversity was described by PCoA based on weighted unifrac distance. Samples with similar community structure tended to gather together, while samples with large community differences were far apart. As shown in the Figure 2, the OTUs distribution of forest land and grassland was partially overlapped, while the cultivated land was relatively independent, which indicates that the OTUs distribution of cultivated land was greatly affected by human management. The difference in OTUs distribution at different distance gradients is: grassland > forest land > cultivated land, which indicates that soil microbial diversity is not only related to land use type, but also closely related to sampling point location. In addition, we found that there was no overlap of OTUs distribution in grassland under different distance gradients, indicating that grassland was more vulnerable to environmental changes and to the distance to the river.

**FIGURE 3:**
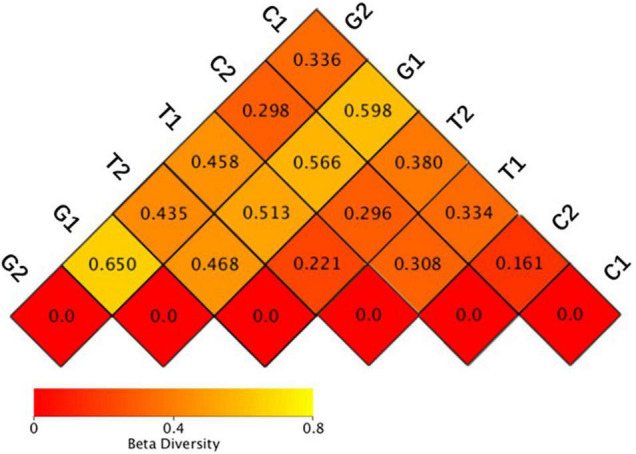
Heatmap of beta diversity index. C1: cultivated land with distance to river from 100 to 200 m; C2: cultivated land with distance to river from 300 to 500 m; G1: grassland with distance to river from 100 to 200 m; G2: grassland with distance to river from 300 to 500 m; T1: forestland with distance to river from 100 to 200 m; T2: forestland with distance to river from 300 to 500 m.

The weighted unifrac distance only considered whether the OTUs is present. If OTUs were the same in two sample groups, their β-diversity differences were minimal. Weighted unifrac distance considered both OTUs presence and OTUs abundance. Therefore, the weighted unifrac distance was selected to comprehensively evaluate the β-diversity, and the distance matrix heatmap was drawn to measure the dissimilarity coefficient between the two samples. The smaller the dissimilarity coefficient was, the smaller the difference of OTUs diversity was. As shown in Figure 3, the dissimilarity coefficient of OTUs diversity between groups in grassland was 0.650 (G1–G2), indicating that the microbial community structure of grassland differed greatly at different distance gradients, which was consistent with the results of α-diversity. With the decrease of distance from the river, the dissimilarity coefficient of OTUs diversity between grassland and cultivated land increased significantly under different distance gradients, which were 0.298 (G2–C2) and 0.598 (G1–C1), respectively, indicating that the difference of microbial community diversity between grassland and cultivated land gradually increased. The AMOVA results showed that there were significant differences among different land uses (Table 3).

**TABLE 3:** Analysis of differences between groups based on the analysis of molecular variance (AMOVA).

vs_group	SS	df	MS	*F*s	*p*-value
T1–T2	0.29 (7.36)	1(35)	0.29 (0.21)	1.38	0.114
C1–C2	0.18 (5.43)	1(38)	0.18 (0.14)	1.29	0.226
G1–G2	1.17 (8.49)	1(22)	1.17 (0.39)	3.03	0.005**
C1–T1	0.84 (4.91)	1(36)	0.84 (0.14)	6.13	< 0.001**
C1–G1	2.13 (9.87)	1(36)	2.13 (0.27)	7.76	< 0.001**
G1–T1	1.49 (10.26)	1(34)	1.49 (0.30)	4.94	< 0.001**
C2–T2	0.56 (7.88)	1(37)	0.56 (0.21)	2.64	< 0.001**
C2–G2	0.38 (4.01)	1(24)	0.38 (0.17)	2.28	0.018*
G2–T2	0.63 (5.60)	1(23)	0.63 (0.24)	2.61	0.006**

SS, sum of squares of deviation; df, degree of freedom; MS, mean square, i.e., SS/DF; Fs: F test value. The level of significance difference is shown as stars; no “*” means no significant difference between the two groups means (p ≥ 0.05); “*” indicates a significant difference between the two groups (p < 0.05); “**” indicates a very significant difference between the two groups (p < 0.01). The values in the brackets are the values corresponding to the residuals. C1: cultivated land with distance to river from 100 to 200 m; C2: cultivated land with distance to river from 300 to 500 m; G1: grassland with distance to river from 100 to 200 m; G2: grassland with distance to river from 300 to 500 m; T1: forestland with distance to river from 100 to 200 m; T2: forestland with distance to river from 300 to 500 m.

### Composition Characteristics of Soil Microbial Community Under Different Land Use Patterns

**FIGURE 4:**
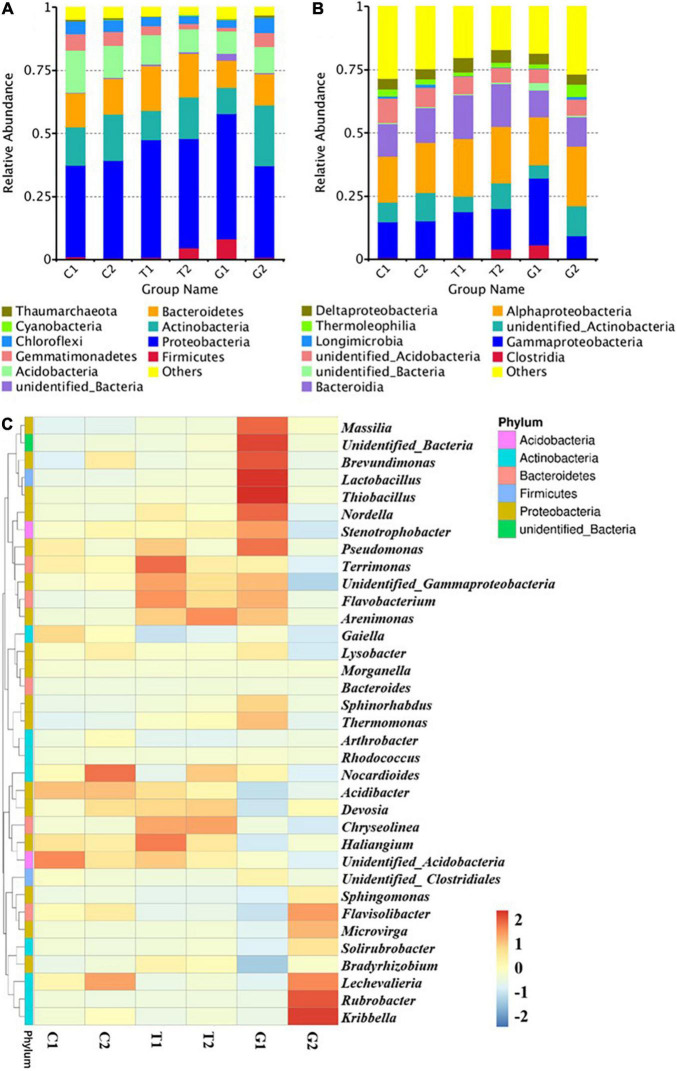
Microbial community composition at different levels: **(A)** phylum, **(B)** class, and **(C)** classified genus (top 35). C1: cultivated land with distance to river from 100 to 200 m; C2: cultivated land with distance to river from 300 to 500 m; G1: grassland with distance to river from 100 to 200 m; G2: grassland with distance to river from 300 to 500 m; T1: forestland with distance to river from 100 to 200 m; T2: forestland with distance to river from 300 to 500 m.

The top 10 phyla with high abundance of known sequences at the level of phylum were Firmicutes, Proteobacteria, Actinobacteria, Bacteroidetes, Acidobacteria, Gemmatimonadetes, Chloroflexi, Cyanobacteria, and Thaumarcharota (Figure 4A), and the phyla with relative abundance of less than 1% in the sample were classified as “others”. At the phylum level, Proteobacteria had the highest abundance, followed by Actinobacteria, Bacteroidetes, and Acidobacteria, and their relative abundance accounted for more than 70% of the whole bacterial population. In G1 and T2, the relative abundance of Firmicutes was higher than that of other groups. The most abundant class was Alphaproteobacteria, followed by Gammaproteobacteria, Bacteroidia, and Actinobacteria (Figure 4B). The abundance of Clostridia was significantly higher than that of other groups in T2 and G1, indicating that Clostridia dominated the Firmicutes and affected the soil microbial structure and abundance.

The top 35 genera in abundance were selected, and the heat map was drawn by clustering from both genera and samples, so as to visually show the aggregation of species in samples. Genus level analysis (Figure 4C) showed that bacteria genera in grassland were significantly different from other groups, and there were different dominant genera under different distance gradients. The representative bacteria genera of G1 were *Massilia*, *Brevundimonas*, *Lactobacillus*, and *Thiobacillus*, and the representative bacteria genera of G2 were *Rubrobacter* and *Kribbella*. Bacteria with more abundance in cultivated land included *Nocardioides* and *Lechevalieria*, while in forestland included *Arenimonas* and *Chryseolinea*. Some bacteria genera commonly existed in each component, such as *Haliangium*, *Stenotrophobacter*, *Terrimonas*, and *Acidibacter*. In general, the compositions of phyla, classes and genera of different components of bacteria and archaea were similar, but the relative abundance showed change differences distance to river, especially for grassland. In the grassland near the river, not only the dominant taxa changed, but also some dominant genera with high abundance appeared.

**FIGURE 5:**
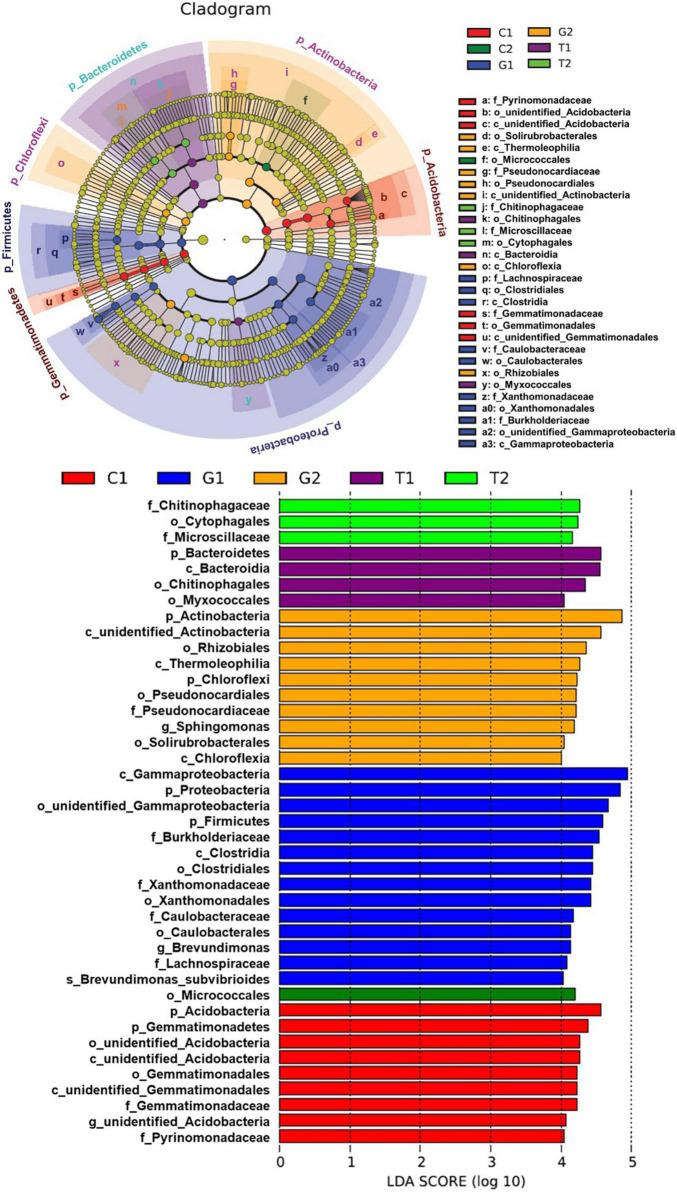
Linear discriminant analysis (LDA) effect size (LefSe) analysis of soil microbial abundance. C1: cultivated land with distance to river from 100 to 200 m; C2: cultivated land with distance to river from 300 to 500 m; G1: grassland with distance to river from 100 to 200 m; G2: grassland with distance to river from 300 to 500 m; T1: forestland with distance to river from 100 to 200 m; T2: forestland with distance to river from 300 to 500 m.

The LefSe was used to identify the species with significant differences in abundance in each group, namely, biomarkers. As can be seen from Figure 5, total of 41 bacterial branches had significant differences, and the LDA threshold was 4.0. The different biomarkers were abundant in grassland soil, suggesting that the structure and diversity of soil microorganisms in grassland were significantly different compared with other land uses. Moreover, it was worth noting that biomarkers in cultivated land decreased significantly with the increase of the distance from the river, and Micrococcales was the only biomarker in C2. The distribution of biomarkers at the phylum level was analyzed by cladogram. At the phylum level, the grassland soil microorganisms were mainly enriched in Proteobacteria, Firmicutes, and Actinobacteria (from class to family). The soil microorganisms in cultivated land were mainly concentrated in Acidobacteria and Gemmatimonadetes (from class to family), and a small part were in Actinobacteria (order). Soil microorganisms in forestland were mainly enriched in Bacteroidetes (from class to family) and a few in Proteobacteria (order).

### Relationship Between Soil Physicochemical Properties and Microbial Communities

**FIGURE 6:**
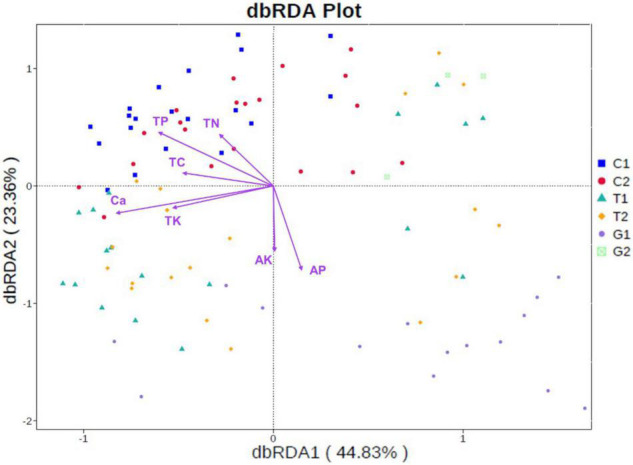
Distance-based redundancy analysis (db-RDA) of soil physicochemical properties with microbial community at phylum level. C1: cultivated land with distance to river from 100 to 200 m; C2: cultivated land with distance to river from 300 to 500 m; G1: grassland with distance to river from 100 to 200 m; G2: grassland with distance to river from 300 to 500 m; T1: forestland with distance to river from 100 to 200 m; T2: forestland with distance to river from 300 to 500 m.

As shown in the Figure 6, the first and second ordination axes of db-RDA respectively explained 44.83 and 23.36% of the microbial community changes. Soil microorganisms in cultivated land gathered in the first and second quadrants, which were obviously positively correlated with TP, and also have a certain correlation with TC and TN. The microbial communities in forestland were mainly distributed in the first and third quadrants, which were mainly restricted by the Ca and TK. The distribution of grassland microbial community was relatively scattered, and the distribution varies greatly under different distance gradients, which was affected by AP and AK. In general, the soil nutrients had a great influence on the microbial community of cultivated land, which was partly related to the application of fertilizer, grazing, and other human activities. Compared with cultivated land and forest land, grassland was less affected by soil nutrients, but it can be seen that its distribution was closely related to the distance from the river.

## Discussion

### Effects of Different Land Use Patterns on Soil Microbial Communities

Land use type is one of the important factors affecting the characteristics of soil microbial communities (Guo and Zhou, 2021). Land use changes can directly alter the soil physicochemical properties and structure, thereby affecting the diversity of soil microbial communities (Liu et al., 2021). In this study, the influence of land use types on the diversity of soil microbial communities showed significant differences under the three different land use modes. Results manifested that the diversity of cultivated land was significantly higher than that of forestland and grassland, consistent with the studies on the Amazon. In recent years, a series of studies on the Amazon have found that the α-diversity of soil microbial communities in cultivated land was significantly higher than that in natural vegetation (Jesus et al., 2009; Rodrigues et al., 2013). However, the result was not consistent with previous studies in the southeastern Tibet (Jiao et al., 2018; Zhang et al., 2020). Jiao et al. (2018) found that the α-diversity of soil bacterial and fungal communities in forest land was significantly higher than that in farmland on the Loess Plateau plot of returning farmland to forest land (Jiao et al., 2018). Zhang et al. (2020) also explored the impact of typical agricultural land use on the characteristics of soil microbial communities in the Nyingchi region of southeastern Tibet, and found that farmland planting significantly reduced soil bacterial and fungal diversity, and its richness and α-diversity were lower than those of natural forests (Zhang et al., 2020). These disparate results suggest that the effects of land use types on soil microbial communities had regional characteristics, which may depend on the type, intensity, and duration of land use.

The different land use types not only affect the diversity of soil microbial communities, but also make differences in the structure of soil microbial communities (Liu et al., 2021). In this study, the dominant phyla of the three different land use types were Proteobacteria, Actinobacteria, Bacteroidetes, and Acidobacteria, which was consistent with previous results (Li et al., 2016; Wu et al., 2017; Gu et al., 2018; Zhou et al., 2019). It can be said that not only in the agro–pastoral areas in high altitude, but also in many areas of the Qinghai-Tibet Plateau, Actinobacteria, Acidobacteria, and Bacteroidetes are the dominant phyla (Wu et al., 2017; Zhou et al., 2019), maintaining a good soil environment for plant growth. These four phyla are the dominant phyla in most soil bacterial communities, but there were some differences in the abundance. The LefSe analysis revealed that there were significant differences in dominant phyla among land use types. It can be found that grassland had more abundant phyla with significant differences than forest land and cultivated land, which indicated that the structure and diversity of soil microorganisms in grassland were significantly different, consistent with the results of previous phyla annotation (Ma et al., 2021). The grassland ecosystem is the “buffer area” of forest land and cultivated land, which can be regarded as an important part of the ecotone in the landscape. In agro–pastoral areas, the grassland ecosystem and cultivated land ecosystem should have mutual influence, but due to the influence of human activities and the natural factors, there are significant differences in soil nutrients and microbial communities (Guo and Zhou, 2021). Therefore, for the method of protecting and improving natural grassland, we should not only adopt rotation grazing and reseeding at the macro level, but also pay attention to improving the nutrients properties of soil, so as to carry out scientific land use management and improve the habitat quality of agro–pastoral areas fundamentally.

### Effects of Soil Nutrients on Microbial Community Diversity

By measuring the soil nutrients, it was found that the soil nutrients such as TC, TN, and TP in grassland were relatively lower than forestland and cultivated land, which was consistent with the results of some previous studies (Shang et al., 2016; Luo et al., 2020). Soil malnutrition is the result of the combination of parent material, topography, climate, vegetation, and other factors (Post et al., 1982). The special terrain and environmental conditions of the Qinghai–Tibet Plateau leaded to the lack of soil nutrients. Especially in some agro–pastoral areas, the dual effects of grazing and farming have made the soil barren and nutrient loss of natural grassland more serious. In addition, without effective attention and protection, it will have a greater negative impact on the ecosystem in the long run (Carrete et al., 2009). Soil nutrients are the main source of plant nutrition, which affects plant and animal composition and microbial community (Zhang et al., 2014). The lack of soil nutrients in the grassland limited the diversity of microbial community. The soil nutrients depend on the transformation and decomposition of various soil microorganisms to form available nutrients that can be directly utilized by plants. As compared with forestland and cultivated land, the content of available nutrients in grassland soil is higher, indicating that there are relatively active soil microbial activities in grassland. Consistent with the results of RDA analysis, except for AK and AP, the soil microorganisms in grassland are less restricted by other measured factors. The microbial communities in cultivated land are closely related to the soil content TC, TN, and TP. A large number of studies have shown that these soil nutrients have a great impact on the diversity and abundance of soil microbial community structure, and play a key role in the change of them (Zhang et al., 2016; Gu et al., 2018; Zeng et al., 2018; Zhou et al., 2019). Moreover, we have also found that the content of Ca and TK is related to the soil microbial community in forest land. The recent studies have shown that the contents of some metal nutrients such as Ca, K, and Fe, are positively related to the microbial community structure, which is also worthy of further investigation in the future (Hamidovic et al., 2020).

Changes in soil moisture and temperature will not only directly affect soil physicochemical properties and nutrient content, but also change the living environment of soil microorganisms, which in turn changes the diversity of the microbial community (Yang et al., 2019). By dividing the land use and the distance gradient from the river, we found that the soil microbial communities in different places showed evident variability. The river nitrogen and phosphorus may be the cause of the differences in nutrient contents along the distance gradient, which affects the activities of soil microorganisms. The microbial diversity of cultivated land and forestland decreased with the increase of distance to river, and the grassland was the opposite. The vegetation types and the human activities may be the reasons for the variability in microbial community structure among different land use types (Jesus et al., 2009; Zhang et al., 2020). The soil nutrients in cultivated land can be improved by humans through fertilization, and nutrients in forest land can be maintained through strong plant roots and a more complex ecosystem structure (Guo and Zhou, 2021; Liu et al., 2021). Thus the cultivated land can maintain a certain soil nutrient content and stability, and the species richness and vegetation density of forestland are higher than those of grassland, which may be the reason why the fluctuation of soil nutrients properties in grassland is larger than that of other two land uses (Liu et al., 2021). Under these conditions, the soil moisture content is particularly important (Zhu et al., 2019). Therefore, water is the main limiting factor in cultivated land and forestland far away from rivers. However, the grassland is different, its soil nutrients properties have not been artificially changed, and the roots of herbaceous plants is not strong enough, coupled with the erosion of the river, it is difficult to have stable soil conditions and rich microbial communities. Thus, there are obvious spatial differences in microbial diversity. In addition, the results of this study for soil nutrients and microbial diversity show that although the nutrients properties of soil have a great contribution to the difference in soil microbial diversity, they cannot be used as a single index for evaluating soil. Especially for grasslands in such special environments as the Qinghai–Tibet Plateau, the differences in microbial diversity are related to soil heterogeneity (such as soil physicochemical properties and soil moisture) and geographical location (Yang et al., 2014). Under the combined influence of these factors, the relationship between soil nutrients and microbial communities is different. For example, in this study, it was found that the microbial diversity of grassland near rivers was lower, while that of cultivated land and forestland was the opposite. This difference indicates that the changes of soil microbial communities are the result of multiple environmental factors.

## Conclusion

In this study, a total of 101 soil plots were collected from 30 sites in the upper reaches of the Yellow River in Qinghai Province. The three land use types of cultivated land, grazing grassland, and plantation forest, the same type was divided into two groups according to the distance from the river. The soil nutrients were determined, and the diversity and composition of the soil microbial community were analyzed. The significant differences in soil nutrients and microbial diversity were found among different land use types and different distance gradients to the river. Among them, the soil nutrients and microbial diversity of grassland have the most significant changes. Also, Proteobacteria, Actinobacteria, and Bacteroidetes were the dominant phyla, and Firmicutes were found to be the main species causing the differences. The RDA results showed that the composition and diversity of soil microbial communities in cultivated land and forest land were mainly related to the contents of soil nutrients, while grassland microbial communities were related with soil available nutrients such as AK and AP, and the community distribution was more dispersed. These results indicated that the grassland ecosystem may be susceptible to the external environment. The LefSe analysis showed that, compared with forestland and cultivated land, the phyla of grassland were more abundant with significant differences, indicating that grassland microbial communities in agro–pastoral areas may play an important role in the energy flow and material cycle of the whole ecosystem. Therefore, the rational protection and utilization of grassland are crucial to maintaining the stability of ecosystem in agro–pastoral areas.

In summary, the agro–pastoral area on the Qinghai–Tibet Plateau, soil properties, plant species, and human activities affect community diversity. The main driving factors for the composition and distribution of microbial communities are different, which may be related to the metabolic methods and survival mechanisms of dominant species. Therefore, more detailed investigations are needed to quantify the contribution of natural and human factors to the diversity and composition of soil microbial communities. An in-depth study of these factors has important theoretical and practical significance for analyzing the complex soil environment, rationally planning land resources, and maintaining the stability of the ecosystem.

## Data Availability Statement

The original contributions presented in the study are included in the article/Supplementary Material, further inquiries can be directed to the corresponding author.

## Author Contributions

SL: writing, reviewing, editing, and validation. YS: editing and methodology. FS: conceptualization, data curation, writing-original draft preparation, investigation, and methodology. YL: investigation and resources. FW and ML: investigation. SD: methodology. All authors contributed to the article and approved the submitted version.

## Conflict of Interest

The authors declare that the research was conducted in the absence of any commercial or financial relationships that could be construed as a potential conflict of interest.

## Publisher’s Note

All claims expressed in this article are solely those of the authors and do not necessarily represent those of their affiliated organizations, or those of the publisher, the editors and the reviewers. Any product that may be evaluated in this article, or claim that may be made by its manufacturer, is not guaranteed or endorsed by the publisher.

## Footnotes

1
http://cutadapt.readthedocs.io/en/stable/

2
http://www.drive5.com/uparse/

3
http://www.arb-silva.de/

4
http://www.drive5.com/muscle/
