# Supplementary material for: Composition and Diversity of Soil Microbial Community Associated With Land Use Types in the Agro–Pastoral Area in the Upper Yellow River Basin
Source: Front Plant Sci. 2022 Apr 25;13:819661. doi: 10.3389/fpls.2022.819661 (PMC9082682; doi:10.3389/fpls.2022.819661)
Supplement: Supplementary file 1 [file Data_Sheet_1.docx]

**Supplementary materials**

**Figure S1**: OTUs venn diagram in different sample groups.


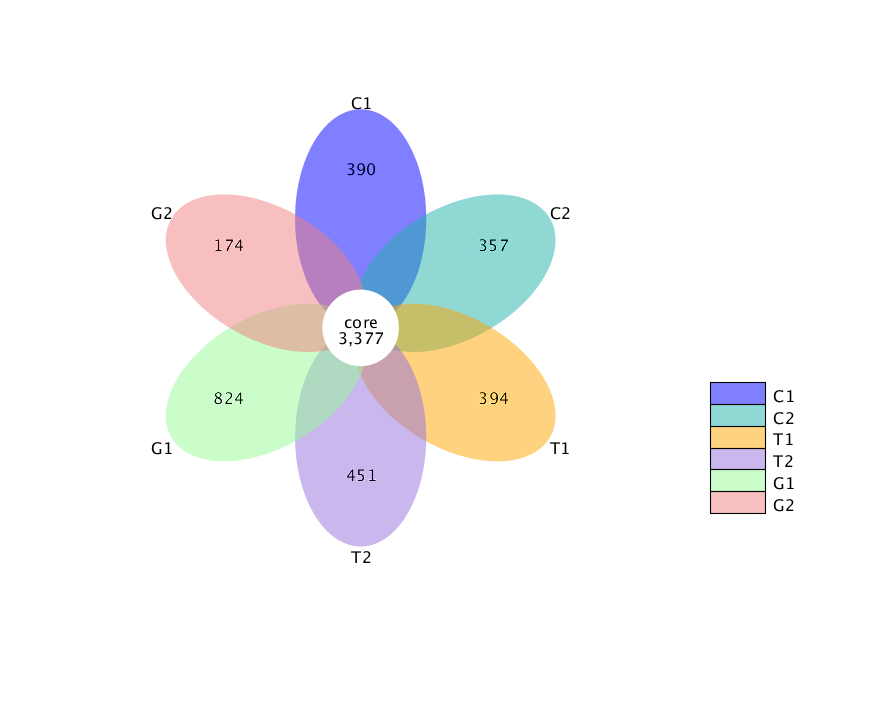


**Figure S1.** OTUs venn diagram in different sample groups.
